# Survival after resection of brain metastasis: impact of synchronous versus metachronous metastatic disease

**DOI:** 10.1007/s11060-023-04242-5

**Published:** 2023-01-25

**Authors:** Anna-Laura Potthoff, Muriel Heimann, Felix Lehmann, Inja Ilic, Daniel Paech, Valeri Borger, Alexander Radbruch, Niklas Schäfer, Patrick Schuss, Hartmut Vatter, Ulrich Herrlinger, Matthias Schneider

**Affiliations:** 1 Department of Neurosurgery, University Hospital, Bonn, Germany; 2 Department of Anesthesiology and Intensive Care Medicine, University Hospital Bonn, Bonn, Germany; 3 Department of Neuroradiology, University Hospital Bonn, Bonn, Germany; 4 Division of Clinical Neuro-Oncology, Department of Neurology, University Hospital Bonn, Bonn, Germany; 5 Present Address: Department of Neurosurgery, BG Klinikum Unfallkrankenhaus Berlin gGmbH, Berlin, Germany

**Keywords:** Surgery for brain metastasis, Synchronous versus metachronous occurrence, Survival

## Abstract

**Purpose:**

Patients with brain metastasis (BM) from solid tumors are in an advanced stage of cancer. BM may occur during a known oncological disease (metachronous BM) or be the primary manifestation of previously unknown cancer (synchronous BM). The time of diagnosis might decisively impact patient prognosis and further treatment stratification. In the present study, we analyzed the prognostic impact of synchronous versus (vs.) metachronous BM occurrence following resection of BM.

**Methods:**

Between 2013 and 2018, 353 patients had undergone surgical therapy for BM at the authors’ neuro-oncological center. Survival stratification calculated from the day of neurosurgical resection was performed for synchronous vs. metachronous BM diagnosis.

**Results:**

Non-small-cell lung carcinoma (NSCLC) was the most common tumor entity of primary site (43%) followed by gastrointestinal cancer (14%) and breast cancer (13%). Synchronous BM occurrence was present in 116 of 353 patients (33%), metachronous BM occurrence was present in 237 of 353 patients (67%). NSCLC was significantly more often diagnosed via resection of the BM (56% synchronous vs. 44% metachronous situation, p = 0.0001). The median overall survival for patients with synchronous BM diagnosis was 12 months (95% confidence interval (CI) 7.5–16.5) compared to 13 months (95% CI 9.6–16.4) for patients with metachronous BM diagnosis (p = 0.97).

**Conclusions:**

The present study indicates that time of BM diagnosis (synchronous vs. metachronous) does not significantly impact patient survival following surgical therapy of BM. These results suggest that the indication for neurosurgical BM resection should be made regardless of a synchronous or a metachronous time of BM occurrence.

**Supplementary Information:**

The online version contains supplementary material available at 10.1007/s11060-023-04242-5.

## Introduction

Approximately 20-40% of all patients with cancer will develop brain metastasis (BM) [2, 3, 13] and neurosurgical resection is an important pillar of treatment in this vulnerable patient clientele with advanced systemically-spread cancer [5]. In addition to providing unequivocal histological diagnosis of the intracranial tumorous lesion, surgical therapy enables metastatic mass reduction resulting in reduced intracranial pressure, prevention of secondary hydrocephalus and improved overall survival (OS) [14, 22]. BM may occur during a known underlying cancer disease (metachronous situation) pretreated with multimodal therapies like radiotherapy, chemotherapy, immunotherapy and specifically-targeted therapies [1]. Compared with this, BM may be the cause of initially presenting symptoms of a previously unknown systemically-spread cancer disease (synchronous situation) [1]. With regard to the presence or the lack of preceding therapies, the time of BM resection dependent on synchronous versus (vs.) metachronous BM occurrence could have a decisive impact on patient prognosis and thus affect surgical and further conservative oncological decision making.

In the present study, we analyzed the prognostic impact of synchronous vs. metachronous BM diagnosis measured from the day of surgical BM resection in patients that had undergone surgery for BM.

## Methods

All patients aged ≥ 18 years (yrs) who had undergone surgery for BM between 01/2013 and 12/2018 at the authors’ neuro-oncological center were registered in a computerized database. Only patients with histopathological proven BM were included in this study. Patients with lost follow-up information regarding the day of death were excluded from further analysis. The study was conducted in accordance with the Declaration of Helsinki and the protocol was approved by the Ethics Committee of the University Hospital Bonn (No. 250/19). Informed consent was not sought as a retrospective study design was chosen.

Pertinent clinical information such as preoperative functional neurological status, comorbidities, radiological features, primary site of cancer and time of diagnosis was assessed. The Karnofsky Performance Score (KPS) was used to classify the patients according to their functional status at admission. Patients were evaluated at admission according to their clinical–functional constitution with KPS ≥ 70% or KPS < 70%, as described previously [20]. The Charlson Comorbidity Index (CCI) was used to evaluate the comorbidity burden of patients prior to surgery. After age adjustment, patients with BM were divided into two groups with CCI < 10 and CCI ≥ 10 as previously described [19]. A weekly tumor board meeting was held at initial presentation and during follow-up to discuss treatment strategies for each patient. Decisions were made by interdisciplinary consensus and, when appropriate, coordinated with the referring physician’s previous therapies [18]. In case of multiple BM, indication for resection was hold for the clinically-manifest lesion, for the prevention of mass effects by the resection of the most prevailing BM and/or to prevent acute tumor-related hydrocephalus.

All patients were divided into two groups for further investigations: Patients with BM as manifestation of a known cancer (metachronous situation) and patients with diagnosis of BM as the first manifestation of an unknown cancer disease (synchronous situation).

OS was defined as the time period from the day of surgery for BM until death or last observation in case the date of death was not known.

### Statistical analysis and graphical illustration

The data collection of this study was performed using the SPSS computer software package for Windows (*Version 27*, IBM Corp., Armonk, NY). Categorical variables were analyzed in contingency tables using the Fisher’s exact test in case of only two variables and using chi-square test if more than two variables were analyzed. To compare data that was not normally distributed, the Mann-Whitney U-test was chosen. OS rates were analyzed by the Kaplan-Meier method using GraphPad Prism software for MacOS (Version 9.4.1, Graphpad Software, Inc., San Diego, California, USA). The Gehan-Breslow-Wilcoxon test was used to compare survival rates. A backward stepwise method was used to construct a multivariate logistic regression model in order to identify predictors of elevated 1-year mortality. Results with p < 0.05 were considered statistically significant. The radar plot was generated using R (Version 3.6.2, Vienna, Austria) as previously described [11].

## Results

### Patient characteristics

Between 2013 and 2018, 388 patients had undergone resection of BM at the neuro-oncological center of the University Hospital Bonn. 35 patients were excluded from further analysis due to the lack of sufficient follow-up information. Therefore, the study cohort was made up of 353 patients with surgically treated BM.

The mean patient age was 64 years (SD ± 12 years) (Table 1). At admission, patients presented with a median KPS of 80 (IQR 70–90) and a median CCI of 11 (IQR 10–12). Most commonly BM originated from NSCLC (n = 153, 43%), followed by gastrointestinal cancer (n = 48, 14%), breast cancer (n = 45, 13%) and melanoma (n = 37, 10%). Supplementary Table S1 depicts comparative hormone receptor status analysis for the tumor of primary site and the BM in patients that had undergone surgery for BM from breast cancer between 2016 and 2019. 112 patients (32%) suffered from multiple BM and 158 patients (45%) presented with additional extracranial metastasis at the time of BM diagnosis. For the overall study cohort, mOS was 13 months (95% CI 10.3–15.7). 204 of 353 patients (58%) died within 1 year after BM resection. For further information on patient characteristics see Table 1.

**Table 1:** Patient characteristics

Baseline characteristics*	n = 353
Median age (yrs, ± SD)	64 ± 12
Female sex	173 (49)
Primary site of cancer	
NSCLC	153 (43)
Gastrointestinal	48 (14)
Breast	45 (13)
Melanoma	37 (10)
Others**	70 (20)
Multiple BM	112 (32)
Extracranial BM	158 (45)
Preoperative KPS (IQR)	80 (70–90)
Median CCI-index (IQR)	11 (10–12)
Median OP duration (min, IQR)	170 (137–214)
1-year mortality	204 (58)
mOS (mo, 95% CI)	13 (10.3–15.7)

*Values represent number of patients unless indicated otherwise (%)

** others: kidney, prostate, thyroid gland and cancer of unknown primary

BM, brain metastasis; CCI, Charlson Comorbidity Index; CI, confidence interval; IQR, interquartile range; KPS, Karnofsky Performance Score; mo, months; min, minutes; mOS, median overall survival; NSCLC, non-small cell lung carcinoma; OP, operation; SD, standard deviation; yrs, years

### Synchronous BM occurrence correlates to NSCLC and multiple BM

Synchronous BM diagnosis was present in 116 of 353 patients (33%) with BM, metachronous BM diagnosis occurred in 237 of 353 patients (67%) with BM (Table 2).

**Table 2:** Patients with surgically treated BM stratified for synchronous vs. metachronous BM occurrence

BM occurrence*	synchronous BM occurrence n = 116	metachronous BM occurrence n = 237	p-value
Median age (yrs, ± SD)	64 ± 11	65 ± 12	0.50
Female sex	58 (50)	115 (49)	0.82
Primary site of cancer			< 0.0001
NSCLC	86 (74)	67 (28)	0.0001
Breast	3 (3)	42 (18)	0.0001
Gastrointestinal	5 (4)	43 (18)	0.0001
Melanoma	7 (7)	28 (12)	0.19
Others	15 (12)	57 (24)	
Multiple BM	49 (42)	63 (27)	0.0035
Extracranial BM	49 (42)	109 (46)	0.65
Preoperative KPS ≥ 70	98 (84)	210 (89)	0.3
Median CCI-index ≥ 10	88 (76)	178 (75)	0.9
Median OP duration (min, IQR)	168 (138–209)	170 (137–215)	0.64
1-year mortality	71 (61)	133 (56)	0.42
mOS (mo, 95% CI)	12 (7.5–16.5)	13 (9.6–16.4)	0.97

*Values represent number of patients unless indicated otherwise (%)

BM, brain metastasis; CCI, Charlson Comorbidity Index; CI, confidence interval; IQR, interquartile range; KPS, Karnofsky Performance Score; mo, months; min, minutes; mOS, median overall survival; NSCLC, non-small cell lung carcinoma; SD, standard deviation; vs., versus; yrs, years

BM from NSCLC was significantly more often detected in the synchronous than in the metachronous situation: in 86 of 153 patients (56%) with BM from NSCLC, NSCLC was diagnosed through resection of the BM, whereas in 67 of 153 patients (44%) with BM from NSCLC, the BM occurred during a known NSCLC disease (p < 0.0001). Compared with this, BM from breast and gastrointestinal cancer entities significantly more often occurred in the metachronous situation (breast cancer: 93% metachronous vs. 7% synchronous, p = 0.0001; gastrointestinal cancer: 90% metachronous vs. 10% synchronous, p = 0.0001) (Table 2).

**Fig. 1:**
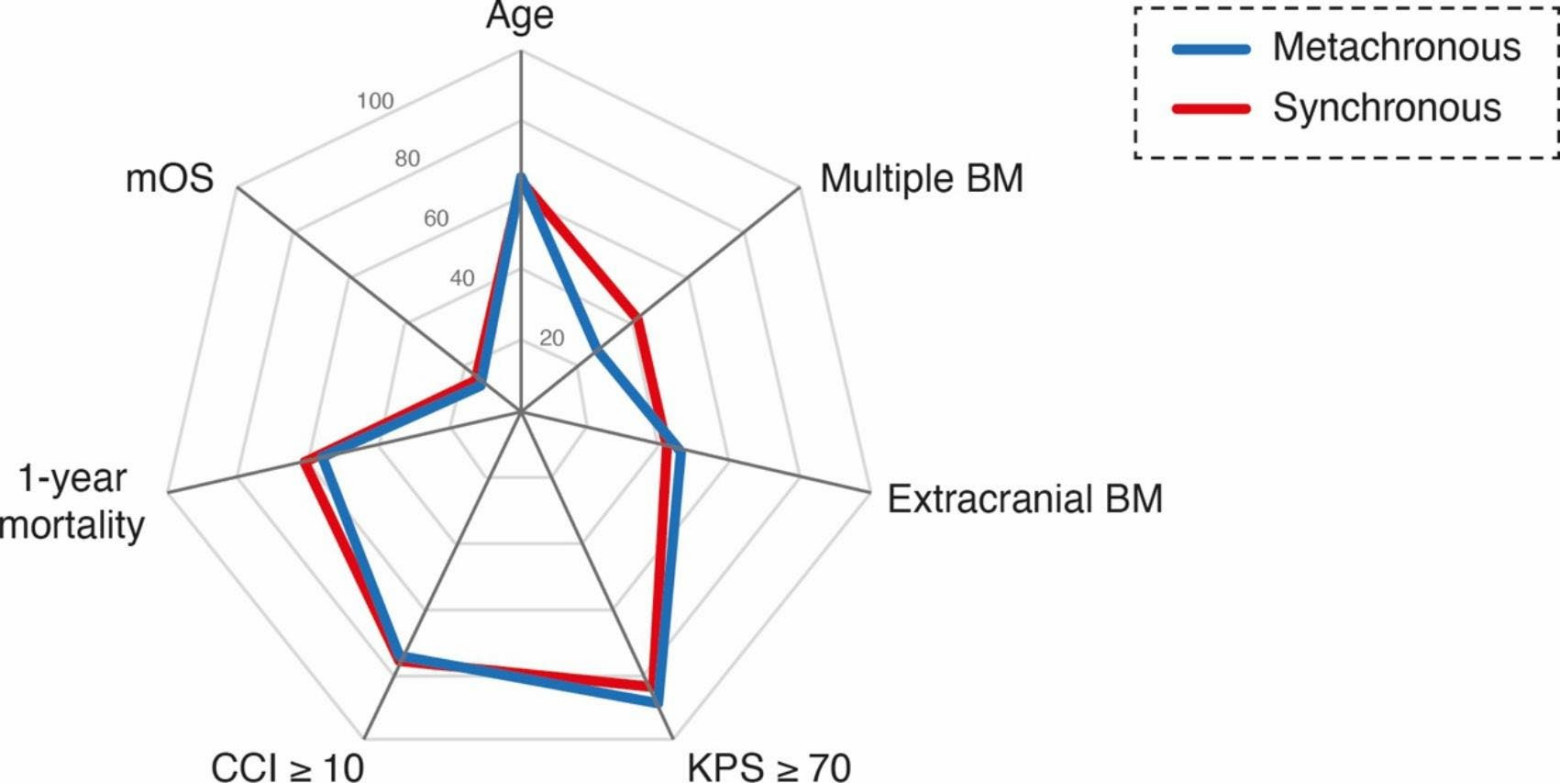
Radar plot depicting patient- and disease-related characteristics dependent on synchronous vs. metachronous BM occurrence in patients with surgically treated BM BM, brain metastasis; CCI, Charlson comorbidity index; KPS, Karnofsky performance score; mOS, median overall survival, vs., versus

49 of 116 patients (42%) in the group of patients with synchronous BM occurrence revealed multiple BM at the day of BM resection compared to 63 of 237 patients (27%) in the group of patients with metachronous BM occurrence (p = 0.0035). Comparative analysis of age, sex, preoperative KPS and CCI as well as the presence of extracranial metastasis at the time of BM diagnosis revealed a homogeneous distribution between the groups of synchronous and metachronous BM occurrence (Table 2; Fig. 1).

### Patient survival following BM resection does not differ between the groups of synchronous and metachronous BM occurrence

**Fig. 2:**
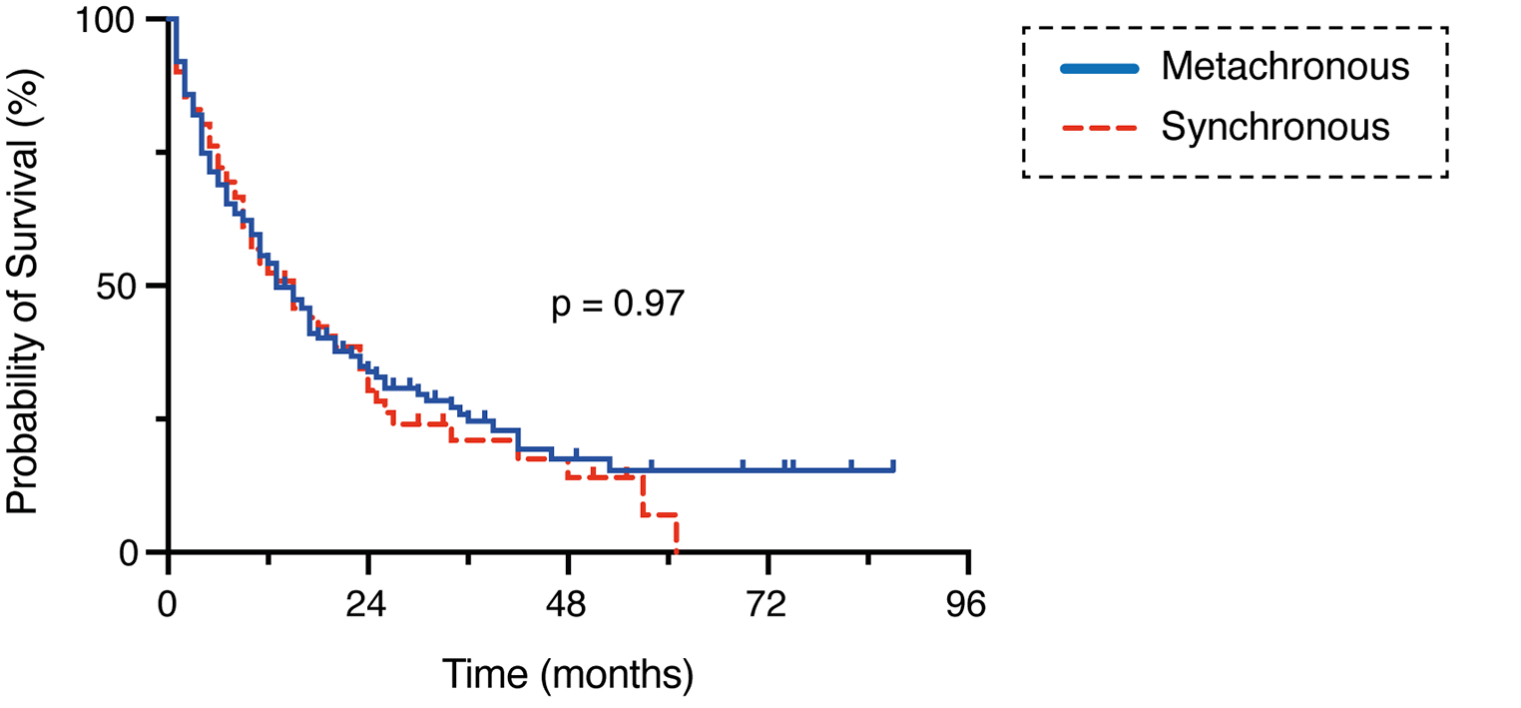
Kaplan-Meier survival analysis dependent on synchronous vs. metachronous BM occurrence BM, brain metastasis; vs., versus

71 of 116 patients (61%) in the group of patients with synchronous BM occurrence died within 1 year after BM resection compared to 133 of 237 patients (56%) in the group of patients with metachronous BM occurrence (p = 0.42) (Table 2; Fig. 2). Patients with synchronous BM diagnosis exhibited a mOS of 12 months compared to 13 months in patients with metachronous BM diagnosis when calculated from the day of BM surgery (p = 0.97) (Fig. 2). Survival analysis for the group of patients with solitary BM revealed a mOS of 15 months (95% CI 11.2–18.8) for the synchronous situation and a mOS of 15 months (95% CI 5.9–24.1) for the metachronous situation (p = 0.7) (Supplementary Figure S1).

Similarly, for the subgroup of patients with NSCLC, 1-year mortality rates and mOS did not significantly differ for the synchronous and metachronous situation: 1-year mortality rate of 59% (synchronous) vs. 48% (metachronous), p = 0.19; mOS of 15 mo (95% CI 7.9–22.1) (synchronous) vs. 23 mo (95% CI 14.7–31.3) (metachronous), p = 0.21 (Supplementary Table S2).

Multivariable regression analysis including age, preoperative KPS, tumor entity NSCLC, multiple BM, additional extracranial metastasis, preoperative comorbidity burden objectified by the CCI and time of BM diagnosis (synchronous vs. metachronous) confirmed the variable ‘time of diagnosis (synchronous vs. metachronous)’ not to constitute an independent negative or positive predictor of increased 1-year mortality in patients that had undergone surgery for BM (adjusted odds ratio (OR) 0.8, 95% CI 0.4–1.3, p = 0.3) (Table 3).

**Table 3:** Multivariable regression analysis for predictors of 1-year mortality

Factors	Adjusted OR	95% CI	p-value
Age ≥ 65 yrs	2.3	1.5–3.6	**< 0.001**
Preoperative KPS < 70	2.5	1.1–5.3	**0.02**
Tumor entity NSCLC	0.8	0.5–1.2	0.3
Multiple BM	2.5	1.1–5.3	**0.001**
Extracranial metastasis	0.9	0.6–1.5	0.8
Preoperative CCI ≥ 10	0.8	0.4–1.5	0.5
*Time of BM diagnosis*	*0.8*	*0.4–1.3*	*0.3*

BM, brain metastasis; CCI, Charlson Comorbidity Index; CI, confidence interval; KPS, Karnofsky Performance Score; NSCLC, non-small cell lung carcinoma; OR, Odds ratio; SD, standard deviation; vs., versus; yrs, years

## Discussion

BM can occur during a known cancer disease (metachronous BM diagnosis) or be the primary manifestation of a previously unknown underlying oncological disease (synchronous BM diagnosis). The present manuscript indicates that time of BM diagnosis does not impact 1-year mortality and patient survival following surgical therapy of BM when measured from the day of BM resection.

NSCLC was the tumor entity that significantly more often had been diagnosed via the BM, whereas BM from gastrointestinal cancer and breast cancer significantly more often occurred in the course of the known underlying cancer disease. Lung cancer is known to cause the highest number of BM with an incidence of BM in literature ranging from 20% up to as high as 56% of all lung cancer patients depending on histological type, epidermal growth factor receptor (EGFR) mutation status and stage of disease [4, 10, 13, 24]. In line with our data, BM in breast cancer are reported to occur in 5-14% of cancer patients therefore ranking among the most prevalent cancer entities that develop BM [3, 15, 17, 21]. Our data additionally reveal that in contrast to BM from breast cancer, BM in NSCLC is more frequently diagnosed in the synchronous than in the metachronous situation. This observation might partly be reasoned in breast cancer pathologies that usually get clinically manifest by breast touch and medical check-up examinations while lung cancer quite commonly remains clinically unmasked until advanced stages of disease [9, 12, 23].

In the present series, the mOS rate of patients with metachronous BM occurrence was 13 months compared to 12 months for patients with synchronous BM occurrence when measured from the day of neurosurgical resection. Though survival analysis in patients with BM is confounded by the underlying heterogeneous tumor landscape, the OS data in our study are within the range of published survival data of patients with BM. According to survival analyses based on a multi-institutional database including over 6.900 patients with newly-diagnosed BM, survival data of patients with NSCLC cover a range of 6 mo to 25 mo dependent on preoperative KPS, age and histopathological subclassification [21]. Corresponding data for breast cancer and melanoma are stated 11 mo to 23 mo and 6 mo to 7 mo [21]. The present data additionally reveal that time of BM diagnosis does not significantly impact patient survival measured from the day of BM resection. These results suggest that the intended prognostic benefit of a surgical therapy in these vulnerable patients [7, 25] is present in both patients with metachronous and synchronous BM occurrence. Therefore, the authors recommend that surgical decision making in these patients with systemically spread cancer should be a process independent of the time of BM diagnosis. These findings also fit with the growing knowledge and clinical experience of histopathological and molecular differences between primary cancer and the corresponding BM which may result in different or additional therapeutic implications [8, 16]. In a study of Brastianos et al. 53% of BM revealed potentially clinically informative alterations that had not been detected in the matched primary-tumor sample suggesting that sequencing of tumor of primary site may miss a substantial number of opportunities for targeted therapy [6]. With regard to the identification of so far undetected molecular targets resulting in the improvement of prognosis via extended treatment options, restriction of BM resection in cancer patients with previous systemic cancer treatment seems unreasonable especially with regard to the survival analysis of the present study. Further multicenter studies will be needed to comprehensively elucidate the overall impact of time of diagnosis in the heterogeneous population of patients with BM.

### Limitations

The present study weakens from several limitations. The study was conducted in a retrospective fashion and patients were not randomized, but treated according to the preferences of the treating physicians. Furthermore, the patient clientele with BM constitutes quite a heterogeneous study population in regard to the underlying cancer disease as well as pretreatment which might lead to relevant unmasked bias in data analysis. Nevertheless, the present study suggests time of BM diagnosis not to significantly impact patient prognosis in BM surgery and thus provides the basis for the initiation of multicenter registries and further studies.

## Conclusions

The present study indicates that time of BM diagnosis (synchronous vs. metachronous) does not significantly impact patient survival when calculated from the day of neurosurgical resection. BM in NSCLC frequently is diagnosed as the first manifestation of the cancer disease (synchronous situation) than it occurs as a long-term consequence of a yet known NSCLC (metachronous situation). These results portend indication for surgical therapy of BM in this vulnerable patient cohort to be a decision independent of the time of BM diagnosis.

## Electronic supplementary material

Below is the link to the electronic supplementary material.

Supplementary Material 1

## Data Availability

Restrictions apply to the availability of these data due to privacy restrictions.
