# Supplementary material for: Survival after resection of brain metastasis: impact of synchronous versus metachronous metastatic disease
Source: J Neurooncol. 2023 Jan 25;161(3):539–45. doi: 10.1007/s11060-023-04242-5 (PMC9992001; doi:10.1007/s11060-023-04242-5)

**Supplementary Table S1: Hormone receptor status evaluation for patients with metachronous BM diagnosis that had undergone resection of BM from mamma carcinoma between 01/2016 and 01/2019***

|  | | **Tumor of primary site**  n=21 | **BM**  n=21 |
| --- | --- | --- | --- |
| Estrogen receptor | |  |  |
|  | positive | 10 (48) | 5 (24) |
|  | negative | 11 (52) | 16 (76) |
| Progesterone receptor | |  |  |
|  | positive | 8 (38) | 3 (14) |
|  | negative | 13 (62) | 18 (86) |
| Her2 | |  |  |
|  | positive | 13 (62) | 14 (67) |
|  | negative | 8 (38) | 7 (33) |

*Values represent number of patients unless indicated otherwise (%)

BM, brain metastasis.

**Supplementary Table S2: Patients with surgically treated BM from NSCLC stratified for synchronous vs. metachronous BM occurrence ***

|  | **synchronous BM occurrence**  **n= 86** | **metachronous BM occurrence**  **n= 67** | **p-value** |
| --- | --- | --- | --- |
| Median age (yrs, ± SD) | 63 ± 10 | 64 ± 8.2 | 0.36 |
| Female sex | 46 (53) | 28 (42) | 0.19 |
| Multiple BM | 37 (43) | 17 (25) | 0.03 |
| Extracranial BM | 36 (42) | 16 (24) | 0.04 |
| Preoperative KPS ≥ 70 | 72 (84) | 59 (88) | 0.49 |
| Median CCI ≥ 10 | 67 (78) | 56 (84) | 0.42 |
| Median OP duration (min, IQR) | 170 (134-218) | 160 (128-199) | 0.29 |
| 1-year mortality | 51 (59) | 32 (48) | 0.19 |
| mOS (months, IQR) | 15 (7.9-22.1) | 23 (14.7-31.3) | 0.21 |

*Values represent number of patients unless indicated otherwise (%)

BM, brain metastasis; CCI, Charlson Comorbidity Index; CI, confidence interval; IQR, interquartile range; KPS, Karnofsky Performance Score; min, minutes; mOS, median overall survival; NSCLC, non-small cell lung carcinoma; SD, standard deviation; vs., versus; yrs, years.

**Supplementary Figure S1:** Kaplan-Meier survival analysis for the subgroup of patients with surgically treated solitary BM dependent on synchronous vs. metachronous BM occurrence

BM, brain metastasis; vs., versus.


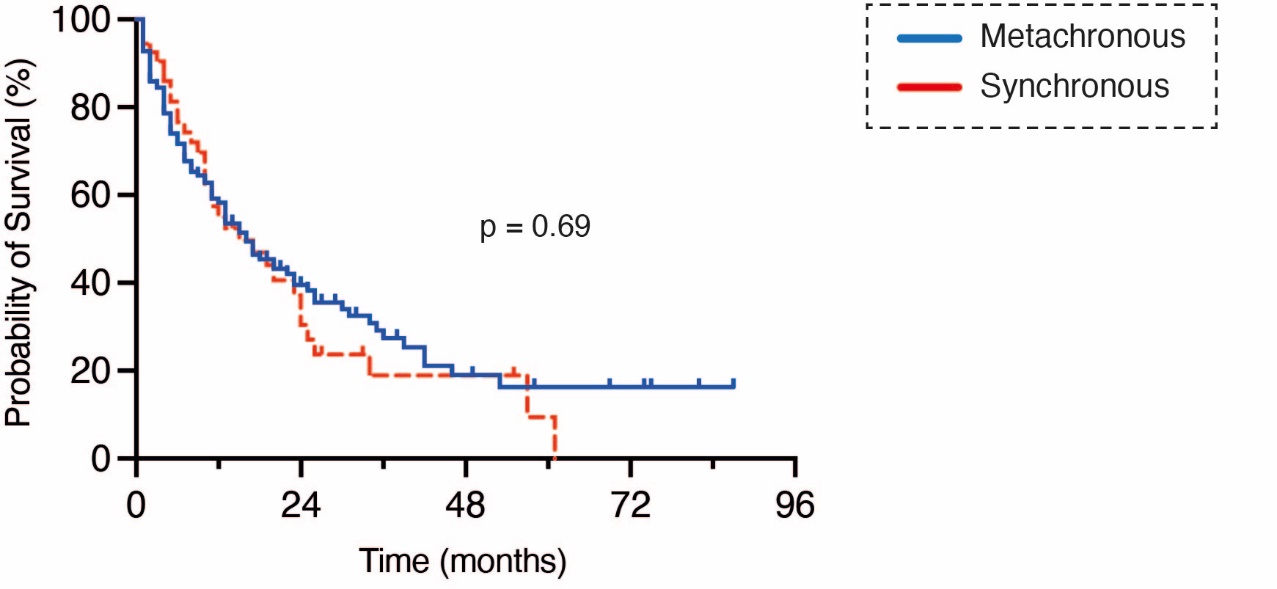

Supplement: Supplementary file 1 — Supplementary Material 1 [file 11060_2023_4242_MOESM1_ESM.docx]
